# Improved single-chain transactivators of the Tet-On gene expression system

**DOI:** 10.1186/1472-6750-7-6

**Published:** 2007-01-19

**Authors:** Xue Zhou, Jori Symons, Rieuwert Hoppes, Christel Krueger, Christian Berens, Wolfgang Hillen, Ben Berkhout, Atze T Das

**Affiliations:** 1 Laboratory of Experimental Virology, Department of Medical Microbiology, Center for Infection and Immunity Amsterdam (CINIMA), Academic Medical Center of the University of Amsterdam, Meibergdreef 15, 1105 AZ Amsterdam, The Netherlands; 2 Lehrstuhl fur Mikrobiologie, Institut fur Mikrobiologie, Biochemie und Genetik, Friedrich-Alexander-Universitat Erlangen-Nurnberg, Staudtstrasse 5, D-91058 Erlangen, Germany

## Abstract

**Background:**

The Tet-Off (tTA) and Tet-On (rtTA) regulatory systems are widely applied to control gene expression in eukaryotes. Both systems are based on the Tet repressor (TetR) from transposon Tn*10*, a dimeric DNA-binding protein that binds to specific operator sequences (*tetO*). To allow the independent regulation of multiple genes, novel Tet systems are being developed that respond to different effectors and bind to different *tetO *sites. To prevent heterodimerization when multiple Tet systems are expressed in the same cell, single-chain variants of the transactivators have been constructed. Unfortunately, the activity of the single-chain rtTA (sc-rtTA) is reduced when compared with the regular rtTA, which might limit its application.

**Results:**

We recently identified amino acid substitutions in rtTA that greatly improved the transcriptional activity and doxycycline-sensitivity of the protein. To test whether we can similarly improve other TetR-based gene regulation systems, we introduced these mutations into tTA and sc-rtTA. Whereas none of the tested mutations improved tTA activity, they did significantly enhance sc-rtTA activity. We thus generated a novel sc-rtTA variant that is almost as active and dox-sensitive as the regular dimeric rtTA. This variant was also less sensitive to interference by co-expressed TetR-based tTS repressor protein and may therefore be more suitable for applications where multiple TetR-based regulatory systems are used.

**Conclusion:**

We developed an improved sc-rtTA variant that may replace regular rtTA in applications where multiple TetR-based regulatory systems are used.

## Background

**Figure 1:**
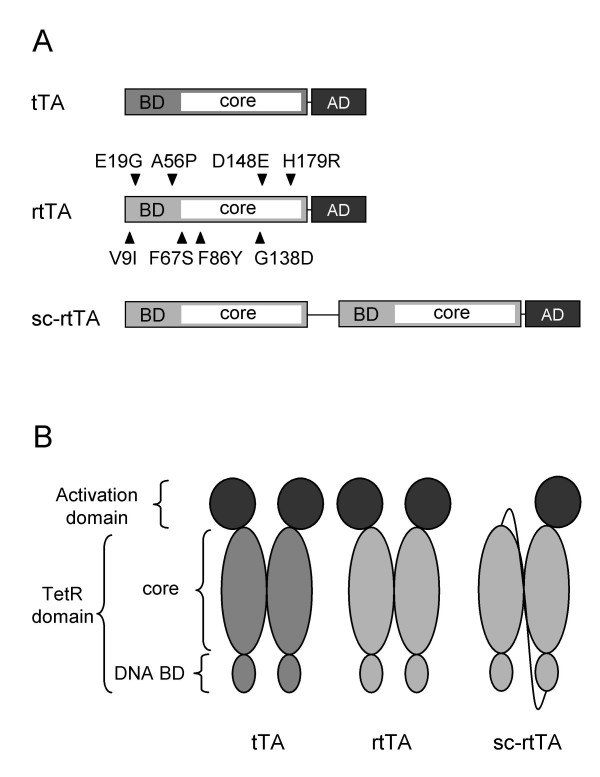
**TetR-based transactivators**. (A) tTA contains the N-terminal transposon Tn*10*-derived Tet repressor and the C-terminal herpes simplex virus VP16-derived activation domain (AD). The TetR part can be subdivided in a DNA-binding domain (BD) and a regulatory core domain (core). rtTA (rtTA2^S^-S2 in ref. [6]) is a tTA variant with four amino acid substitutions in the TetR domain (shown above the protein). The mutations that enhance rtTA activity are also located in the TetR domain (shown under the protein). sc-rtTA is a single-chain version of rtTA that contains two TetR moieties connected head to tail by a peptide linker and a single activation domain at the C-terminal end. (B) Both tTA and rtTA are active as homodimers, whereas sc-rtTA folds intramolecularly.

Inducible gene regulation systems that utilize small, non-toxic effector molecules to control transgene expression in eukaryotic cells and organisms have become invaluable tools in many biological research areas, such as functional genomics and gene therapy. Among the currently used regulatory circuits, the Tet-Off and Tet-On systems based on the tetracycline-resistance determinant from transposon Tn*10 *are the most widely applied and best studied [1-3]. In *Escherichia coli*, the Tet repressor protein (TetR) forms a dimer that binds to the *tet *operator (*tetO*) DNA sequence with high affinity and specificity. Tetracycline (Tc) or its derivative doxycycline (dox) binds to TetR and triggers a conformational change that prevents the protein from binding to *tetO*. Fusion of TetR to the VP16 activation domain of herpes simplex virus resulted in the Tc-responsive transactivator (tTA) which has retained the DNA-binding specificity and effector-inducibility of TetR [4]. In the absence of effector, tTA binds to *tetO *and activates transcription from an appropriately-positioned minimal promoter. Administration of Tc or dox switches gene expression off (Tet-Off system). tTA variants have been isolated that carry amino acid substitutions in the TetR part and exhibit a reverse phenotype [5,6] (Fig. 1). These reverse tTAs (rtTAs) activate gene expression from *tetO*-containing minimal promoters only in the presence of dox (Tet-On system). Both Tet systems have been used to regulate gene expression in a wide variety of applications [2]. TetR has also been fused to the KRAB repression domain of the human Kox1 protein to form a Tc-responsive transsilencer (tTS), which can actively suppress background gene expression from *tetO*-containing minimal promoters in the absence of dox [7]. The combined use of tTS and rtTA has resulted in more stringent dox-control of target genes [8,9]. In such systems, the Tn*10*-derived TetR moiety of tTS was replaced with other natural TetR variants to prevent heterodimerization with rtTA [8-11].

Current research focuses on developing additional TetR-based transregulators that respond to specific Tc-derivatives and recognize distinct operator sequences [12-16]. Such novel Tet systems will allow the independent regulation of multiple (sets of) genes by different effectors [16,17]. Again, the formation of heterodimers has to be prevented. Transfer of the mutations responsible for the altered phenotypes to other natural sequence variants represents one strategy, but such changes can lead to non-functional transregulators [17,18]. As an alternative strategy, single-chain Tet transregulators have recently been developed in which two TetR domains are connected by a flexible peptide linker, and a single functional domain, like an activation, repression or oligomerization domain is fused to the C-terminus [18-20] (Fig. 1). These transregulators fold intramolecularly and do not dimerize with each other. Unfortunately, the single-chain version of rtTA (sc-rtTA) exhibits reduced activity when compared with the dimeric rtTA, and this may restrict its application [18].

We have previously incorporated the rtTA2^S^-S2 gene encoding a second-generation rtTA variant [6] and the *tetO *elements into the HIV-1 genome to control virus replication [21,22]. During culturing of this dox-dependent virus, spontaneous evolution selected for improved virus variants in which the introduced Tet-On system was found to be optimized [23-27]. We have identified several amino acid substitutions that greatly enhance the transcriptional activity and dox-sensitivity of rtTA [24,26]. To test whether these mutations can similarly improve other TetR-based transactivators, they were introduced into tTA and sc-rtTA. Whereas none of these mutations improve tTA activity, they all enhance sc-rtTA activity. The most active sc-rtTA variant that we generated is 30-fold more active than the original sc-rtTA, and almost as active as the regular rtTA.

## Results

### Mutations that enhance rtTA activity do not improve tTA activity

**Figure 2:**
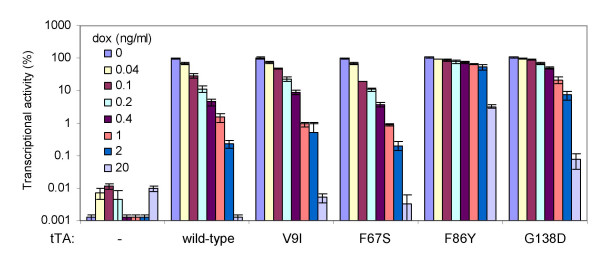
**Mutations that enhance rtTA activity do not improve tTA activity**. The transcriptional activity of tTA variants was measured in HeLa X1/6 cells [28] containing chromosomally integrated copies of the CMV-7*tetO *luciferase reporter construct. Cells were transfected with the tTA expression plasmids indicated or pBluescript (-) as a negative control and a plasmid constitutively expressing *Renilla *luciferase to correct for differences in transfection efficiency. Cells were cultured in the presence of different dox concentrations (0–20 ng/ml). The ratio of the firefly and *Renilla *luciferase activities measured two days after transfection reflects the tTA activity. All values were related to the original (wild-type) tTA activity in the absence of dox, which was arbitrarily set at 100%. Average values of two transfections are shown with the error bars indicating the standard deviations.

The transactivators tTA and rtTA are fusion proteins containing an N-terminal domain consisting of the TetR protein from transposon Tn*10 *and a C-terminal herpes simplex virus VP16-derived activation domain. The activation domains are identical in both proteins, whereas the TetR domains differ from each other by four amino acids (Fig. 1A). We have used viral evolution to optimize the function of the Tet-On system, and identified several amino acid substitutions in the rtTA protein (rtTA2^S^-S2 [6]) that greatly enhance its transcriptional activity and dox-sensitivity [24,26] (Fig. 1A). To test whether these amino acid substitutions could similarly improve tTA, we introduced the V9I, F67S, F86Y and G138D mutations and compared the activity of these variants with that of the wild-type tTA (tTA2^S ^[6]). The tTA expression plasmids were transfected into HeLa X1/6 cells containing chromosomally integrated copies of the CMV-7*tetO *luciferase-reporter construct [28]. Transfected cells were cultured at different dox concentrations for two days. We subsequently determined the intracellular luciferase level, which reflects tTA activity (Fig. 2). Wild-type tTA shows high transcriptional activity without dox, and its activity is gradually reduced with increasing dox concentrations. For example, the activity is reduced to 30% at 0.1 ng/ml dox, and 0.2% residual activity is observed at 2 ng/ml dox. All variants show a transcriptional activity similar to wild-type tTA in the absence of dox, but they respond differentially to the presence of dox. While the V9I and F67S variants are similarly inhibited by dox as wild-type tTA, the F86Y and G138D variants are less efficiently inhibited. The F86Y and G138D variants show more than 80% residual activity at 0.1 ng/ml dox, and 50% and 7% residual activity, respectively, at 2 ng/ml dox. These results demonstrate that mutations beneficial for rtTA do not necessarily improve tTA activity. In fact, the F86Y and G138D mutations reduce the dox-sensitivity of tTA.

### Mutations observed in rtTA can improve sc-rtTA activity

**Figure 3:**
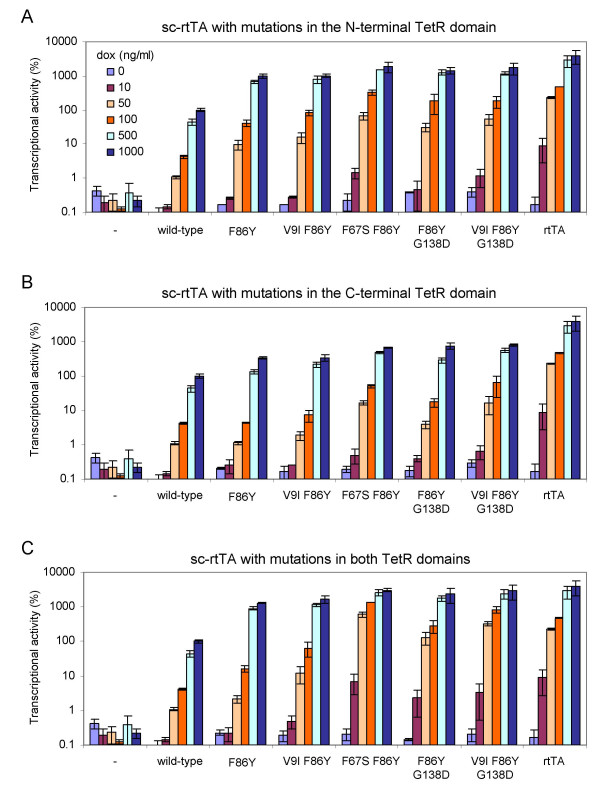
**Mutations observed in rtTA can improve sc-rtTA activity**. The transcriptional activity of rtTA and sc-rtTA was measured in HeLa X1/6 cells, see Fig. 2 for details. Cells were cultured in the presence of different dox concentrations (0–1000 ng/ml). All values were related to the original (wild-type) sc-rtTA activity at 1000 ng/ml dox, which was arbitrarily set at 100%. Average values of two transfections are plotted with the error bars indicating the standard deviations.

In sc-rtTA, two TetR moieties are connected head to tail by a peptide linker, and a single activation domain is fused to the C-terminus (Fig. 1). The mutations that did improve rtTA activity are all positioned within the TetR part of the transactivator. To test whether these beneficial mutations can also improve the activity and dox-sensitivity of sc-rtTA, we introduced them into a single (N- or C-terminal) or both TetR moieties. The activity of these three sets of variants was analyzed in HeLa X1/6 cells and compared with the activity of the wild-type sc-rtTA (sc-rtTA2-S2) and the regular rtTA (rtTA2^S^-S2) (Fig. 3). Both the wild-type sc-rtTA and the regular rtTA show no background activity without dox, and their activity gradually increases with increasing dox levels. However, the induced activity of sc-rtTA is lower than that of rtTA at all dox concentrations tested. For example, sc-rtTA is about 40-fold less active than rtTA at 1000 ng/ml dox. Introduction of the F86Y mutation in the N-terminal TetR domain increased sc-rtTA activity ~10-fold at all dox levels, but did not affect background activity (Fig. 3A). The additional introduction of the V9I mutation into the F86Y variant only marginally improved sc-rtTA activity, whereas the addition of the F67S, G138D, or V9I plus G138D mutations further improved sc-rtTA activity ~2-fold at all dox levels. The background activity of these variants was not increased.

Similar results were obtained upon introduction of the mutations in the C-terminal TetR part (Fig. 3B). However, none of these variants are as active as their counterparts with mutations in the N-terminal TetR moiety. The F86Y mutation increased sc-rtTA activity ~3-fold, and the addition of the F67S, G138D, or V9I plus G138D mutations further increased activity ~2-fold. These results demonstrate that the activity of sc-rtTA can be improved by mutations in either TetR moiety, but mutations in the N-terminal TetR domain have a larger impact on activity than the same mutations in the C-terminal domain.

**Figure 4:**
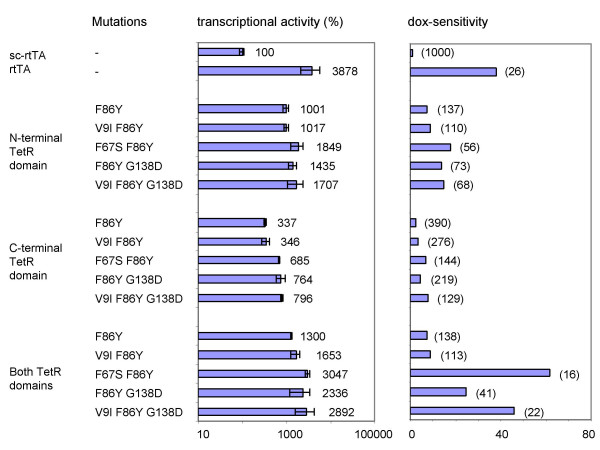
**Transcriptional activity and dox-sensitivity of the sc-rtTA variants**. Calculation is based on the data of Fig. 3. Transcriptional activity observed at 1000 ng/ml dox is shown as average value of two transfections with error bars indicating the standard deviations (wild-type sc-rtTA activity set at 100%). Dox-sensitivity is compared with the wild-type sc-rtTA (set at 1). For each variant, the dox concentration (ng/ml) that results in an activity comparable to that of the wild-type sc-rtTA activity at 1000 ng/ml dox is indicated between brackets.

Introduction of the mutations in both TetR parts resulted in the most active sc-rtTA variants (Fig. 3C). All these variants demonstrate a higher transcriptional activity than the corresponding variants with mutations in only one of the two TetR moieties. For instance, the sc-rtTA with the F86Y mutation in both TetR moieties is ~13-fold more active than wild-type sc-rtTA at 1000 ng/ml dox, whereas the same mutation in the N-terminal or in the C-terminal TetR domain increased sc-rtTA activity ~10-fold and ~3-fold, respectively. The variants containing the F67S, G138D, or V9I plus G138D mutations in addition to the F86Y mutation in both TetR moieties demonstrate an up to 30-fold increased activity at 1000 ng/ml dox. Moreover, these variants are particularly more sensitive to low dox levels. To compare the dox-sensitivity of the sc-rtTA variants, we calculated the dox concentration that each variant needs to reach an activity similar to that of the wild-type sc-rtTA at 1000 ng/ml dox (set at 100% in Fig. 4). The lower the required dox concentration, the more sensitive the variant is toward dox. For example, the variant carrying the F67S and F86Y mutations in both TetR moieties requires only 16 ng/ml dox to reach this 100% activity level, which reflects a 62-fold higher dox-sensitivity than the wild-type sc-rtTA. This variant thereby becomes the most dox-sensitive and most active sc-rtTA (30-fold more active than the wild-type, Fig. 4). In fact, both the transcriptional activity and dox-sensitivity of this sc-rtTA variant are similar to that of the original dimeric rtTA.

### New sc-rtTA variants improve dox-control in combined activation/repression system

**Figure 5:**
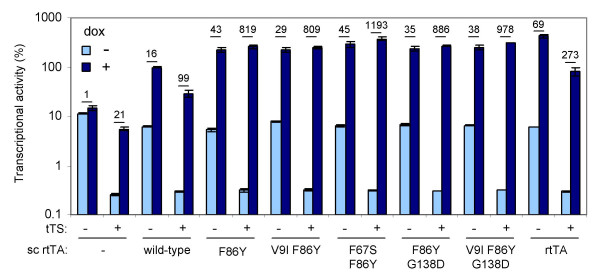
**Combinatorial regulation of gene expression by sc-rtTA and tTS**. Transcription from the CMV-7*tetO *luciferase reporter construct was measured upon transfection of C33A cells with the indicated rtTA, sc-rtTA (wild-type or with mutations in both TetR domains) and tTS expression plasmids or pBluescript (-) as a negative control, and a plasmid constitutively expressing *Renilla *luciferase to correct for differences in transfection efficiency. Cells were cultured with (1000 ng/ml) or without dox. The ratio of the firefly and *Renilla *luciferase activities measured two days after transfection reflects the rtTA activity. All values were related to the original (wild-type) sc-rtTA activity at 1000 ng/ml dox, which was arbitrarily set at 100%. Average values of three transfections are plotted with the error bars indicating the standard deviations. The ratio between the induced (+ dox) and uninduced (no dox) promoter activity (fold induction) is indicated on top of the bars.

In the HeLa X1/6 cell line, the CMV-7*tetO *luciferase-reporter construct is stably integrated into the chromosome and shows no background activity in the absence of dox. However, when this reporter construct is transiently transfected into cells, multiple copies will be present, which may increase background activity and thus reduce inducibility (*i.e*. reduce the ratio between dox-induced and uninduced promoter activity). We therefore tested the new sc-rtTA variants with mutations in both TetR domains upon transient cotransfection of C33A cells with the CMV-7*tetO *luciferase-reporter construct (Fig. 5). As observed with HeLa X1/6 cells, the dox-induced activity of the new sc-rtTA variants in C33A cells is higher than that of the wild-type sc-rtTA, and the new variants are almost as active as the regular rtTA, while the background activity is not affected by the introduced mutations. However, this background promoter activity is relatively high (~7%) and as a consequence the inducibility is low with all rtTA and sc-rtTA variants (16–69 fold).

Since the background expression from *tetO*-controlled minimal promoters can be actively suppressed by tTS [8,9], we cotransfected C33A cells with a tTS expression plasmid (tTS^S ^[18]). The presence of tTS indeed reduced the background activity more than 20-fold (~0.3% residual activity in the absence of dox; Fig. 5). Administration of dox inactivates tTS and activates the (sc-)rtTAs. The dox-induced activity of rtTA and the wild-type sc-rtTA was however ~5-fold reduced by the presence of tTS. This may be due to heterodimerization between the tTS and rtTA or sc-rtTA molecules [29]. The dox-induced activity of the new sc-rtTA variants was not affected by the presence of tTS. The low background and high dox-induced activity observed when combining the new sc-rtTA variants with tTS results in a high inducibility (809–1193 fold), whereas combining the wild-type sc-rtTA or regular rtTA with tTS yields a much lower inducibility (99 and 273 fold, respectively). The highest inducibilty is obtained with the F67S/F86Y-mutated sc-rtTA variant, which was also the most active and dox-sensitive variant when tested without tTS.

## Discussion

We recently identified amino acid substitutions in rtTA that greatly improve the transcriptional activity and dox-sensitivity of the transactivator [24]. In this study, we tested whether these mutations similarly affect other TetR-based transactivators. The results demonstrate that whereas none of the mutations that we tested improved tTA activity, all mutations did enhance sc-rtTA activity significantly. The F67S/F86Y-mutated sc-rtTA proved to be the most active and dox-sensitive single-chain variant and was as active and dox-sensitive as the original dimeric rtTA.

Combining the rtTA and sc-rtTA variants with the repressor tTS reduced the background activity of the CMV-7*tetO *promoter. The dox-induced activity of the regular rtTA and the wild-type sc-rtTA was also reduced in the presence of tTS. This may be due to heterodimerization between the tTS and rtTA or sc-rtTA molecules, which are all based on the TetR class B from transposon Tn*10*. For sc-TetR and for sc-tTS, heterodimer formation is not observed [18], but this was not tested for sc-rtTA variants. The dox-induced activity of the new sc-rtTA variants was not affected by the presence of tTS. The introduced mutations may have modified the sc-rtTA dimerization surface indirectly, as none of the altered amino acids contribute directly to dimerization, or they may have improved intramolecular folding, thus preventing binding to tTS. This resistance to interference by other TetR-based transregulators makes the new sc-rtTA variants very useful in applications where multiple TetR-based regulatory systems are used simultaneously.

The fact that the mutations do not similarly affect tTA and rtTA is probably due to structural differences between the two proteins. In tTA, the natural TetR conformation and dox-response are preserved. In rtTA, the TetR part contains four amino acid substitutions that cause a reciprocal dox-response. These amino acid substitutions do not only confer the reverse phenotype, but also result in a 100-fold reduced dox-sensitivity [3]. This suggests that the structure of the mutated TetR is not optimal for the function of rtTA. The mutations identified in the viral evolution experiments may compensate for such unwanted tTA to rtTA structural changes, and thus specifically enhance rtTA activity. While the V9I mutation in the DNA-binding domain is likely to increase the dox-induced binding affinity of rtTA for the *tetO *site, the F67S, F86Y, and G138D mutations positioned in the regulatory core domain may increase the binding affinity for dox or facilitate the structural transition to the DNA-binding form [24,26]. In contrast, the structure of TetR in tTA is the product of natural evolution of bacterial Tc-resistance regulation. Apparently, introduction of the selected mutations into this already-optimized sequence has no (V9I and F67S) or a detrimental effect (F86Y and G138D). The reduced dox-responsiveness of the F86Y and G138D mutated tTAs is in agreement with previous TetR-mutation studies demonstrating that amino acid substitutions at positions 86 and 138 can cause an induction-deficient phenotype [30].

Both the transactivators rtTA and sc-rtTA are activated by dox. However, the induced activity of sc-rtTA is reduced when compared with rtTA [18]. This is probably due to structural differences between these two proteins. First, sc-rtTA contains only one activation domain per active molecule, whereas the rtTA dimer contains two activation domains. Second, the two TetR parts of sc-rtTA are connected by a peptide linker. Although the linker was designed to be flexible, it may still interfere with the function of the TetR moieties. Our results demonstrate that the sc-rtTA activity can be significantly improved by introduction of mutations that enhanced rtTA activity, suggesting that sc-rtTA folds very similarly to rtTA.

The most active sc-rtTA variant in this study was obtained by introducing beneficial mutations in both TetR domains. The single-chain characteristic of sc-rtTA allows the modification of a single TetR part. We demonstrated that sc-rtTA can be improved by mutations in only one of the TetR moieties, and that the introduction of the mutations in the second TetR domain has an additive effect. This suggests that dox binding to one of the effector-binding pockets does not affect binding to the second one, which is in agreement with published data showing that the binding of two Tc molecules to a TetR dimer occurs without significant cooperativity [31]. The sc-rtTA variants with beneficial mutations in the N-terminal TetR domain appeared to be more active than the variants with the same mutations in the C-terminal TetR part. This may indicate that the induction of the C-terminal TetR domain contributes less to the induced activity of sc-rtTA. Possibly, it binds less efficiently to the *tetO *site due to the close proximity of the DNA-binding domain to the linker peptide.

## Conclusion

We generated a novel sc-rtTA variant that is more active and more dox-sensitive than the original sc-rtTA, and does not show any background activity in the absence of dox. This novel sc-rtTA is almost as active and dox-sensitive as the regular dimeric rtTA but is less sensitive to interference by other TetR-based transregulators, and may therefore be more suitable for applications where multiple TetR-based regulatory systems are used.

## Methods

### Construction of tTA variants

The plasmid pCMV-tTA contains the tTA2^S ^gene cloned in the expression vector pUHD141-1/X [6]. Mutations were introduced in pCMV-tTA by mutagenesis PCR [32] with the mutagenic primers (primer M) tTA-V9I (5'-GGACAAGAGCAAAATCATAAACTCTGCTCTGGA-3', mismatching nucleotide underlined), tTA-F67S (5'-CATACCCACTCCTGCCCCCTGGAAGGCGA-3'), tTA-F86Y (5'-GCGGAACAACGCCAAGTCATACCGCTGTGCT-3'), or tTA-G138D (5'-GTCCGCCGTGGACCACTTTACACTGGGCT-3') and the primers 5'-TGGAGACGCCATCCACGCT-3' (primer 1), 5'-TGAAATCGAGTTTCTCCAGGCCACATATGA-3' (primer 2), and 5'-TCACTGCATTCTAGTTGTGGT-3' (primer 3). Briefly, PCR reactions were performed with primer M plus primer 3, and with primer 1 plus primer 2. The PCR products were purified, mixed, and PCR amplified with primers 1 and 3 (see ref. [32] for details). The resulting mutated tTA genes were cloned as EcoRI-BamHI fragments into pCMV-tTA. All constructs were verified by sequence analysis.

### Construction of sc-rtTA variants

The plasmids pCMV-rtTA and pCMV-sc-rtTA contain the rtTA2^S^-S2 and sc-rtTA2-S2 genes, respectively, cloned in the expression vector pUHD141-1/X [6,18]. The sc-rtTA gene contains two TetR moieties and a single activation domain. To introduce mutations into the N-terminal *tetR *gene (with synthetic humanized codon usage), the EcoRI-BfuAI fragment of pCMV-sc-rtTA was replaced with the corresponding fragment of the appropriate pCMV-rtTA plasmid [26]. Mutations were introduced into the C-terminal *tetR *gene (with original bacterial codon usage) by mutagenesis PCR [32] on pCMV-sc-rtTA with the mutagenic primers (primer M) sc-rtTA-V9I (5'-GGCTCTAGATCTCGTTTAGATAAAAGTAAAATCATTAACAGCGCA-3'), sc-rtTA-F67S (5'-AGGCACCATACTCACTCTTGCCCTTTA-3'), sc-rtTA-F86Y (5'-AACGCTAAAAGTTATAGATGTGCT-3'), or sc-rtTA-G138D (5'-CAGCGCTGTGGACCACTTTACTTTA-3') and the primers 5'-TAATCATATGTGGCCTGGAGAA-3' (primer 1), 5'-AGGCGTATTGATCAATTCAAGGCCGAATAAG-3' (primer 2), and 5'-TCACTGCATTCTAGTTGTGGT-3' (primer 3). The final PCR products were digested with BglII and SmaI and used to replace the corresponding fragment of pCMV-sc-rtTA. All constructs were verified by sequence analysis.

### Cell culture and (r)tTA activity assay

The activity of tTA, tTS, rtTA and sc-rtTA was assayed in C33A cells (ATCC HTB31) [33] and HeLa X1/6 cells [28], which are HeLa-derived cells containing chromosomally integrated copies of the CMV-7*tetO *luciferase reporter construct pUHC13-3 [4]. Cells were cultured at 37°C with 5% CO_2 _in Dulbecco's modified Eagle's medium supplemented with 10% fetal calf serum, minimal essential medium nonessential amino acids, penicillin (100 U/ml), and streptomycin (100 μg/ml). Cells were grown in 2-cm^2 ^wells to 60% confluency and transfected with the pCMV-tTA, pCMV-tTS (pWHE122sB, [18]), pCMV-rtTA or pCMV-sc-rtTA expression plasmids and the plasmid pRL-CMV (Promega) by the calcium phosphate precipitation method. pRL-CMV expresses *Renilla *luciferase from the CMV promoter and was used as an internal control to allow correction for differences in transfection efficiency. 1 μg of the DNA mixture in 15 μl water was mixed with 25 μl of 50 mM HEPES (pH 7.1)-250 mM NaCl-1.5 mM Na_2_HPO_4 _and 10 μl of 0.6 M CaCl_2_, incubated at room temperature for 20 min, and added to the culture medium. The DNA mixture contained 8 ng pCMV-tTA and 2.5 ng pRL-CMV for the tTA activity assay, 20 ng pCMV-sc-rtTA or pCMV-rtTA and 2 ng pRL-CMV for the sc-rtTA or rtTA activity assay in HeLa X1/6 cells, and 5 ng pCMV-sc-rtTA or pCMV-rtTA, 20 ng pCMV-7*tetO*-luciferase (pUHC13-3) [4], 0.5 ng pRL-CMV and 200 ng pCMV-tTS (when indicated) for the sc-rtTA or rtTA activity assay in C33A cells. The DNA mixture was completed to 1 μg with pBluescript as carrier DNA. Cells were cultured after transfection for 48 hours at different dox (D-9891, Sigma) concentrations and then lysed in Passive Lysis Buffer (Promega). Firefly and *Renilla *luciferase activities were determined with the Dual-Luciferase Reporter Assay (Promega). The expression of firefly and *Renilla *luciferase was within the linear range and no squelching effects were observed. The activity of the transactivators was calculated as the ratio of the firefly and *Renilla *luciferase activities, and corrected for between-session variation as described [34].

## Authors' contributions

ATD, BB and XZ designed the experiments. XZ and RH performed the sc-rtTA experiments. JS performed the tTA experiments. XZ analyzed the data and drafted the manuscript. ATD and BB revised the manuscript. CB, CK and WH developed the sc-rtTA system, provided reagents and revised the manuscript.
